# Ultrafast laser ablation simulator using deep neural networks

**DOI:** 10.1038/s41598-022-09870-x

**Published:** 2022-04-07

**Authors:** Shuntaro Tani, Yohei Kobayashi

**Affiliations:** The Institute for Solid State Physics, The University of Tokyo, Kashiwa, Chiba 277-8581 Japan

**Keywords:** Laser material processing, Optical physics

## Abstract

Laser-based material removal, or ablation, using ultrafast pulses enables precision micro-scale processing of almost any material for a wide range of applications and is likely to play a pivotal role in providing mass customization capabilities in future manufacturing. However, optimization of the processing parameters can currently take several weeks because of the absence of an appropriate simulator. The difficulties in realizing such a simulator lie in the multi-scale nature of the relevant processes and the high nonlinearity and irreversibility of these processes, which can differ substantially depending on the target material. Here we show that an ultrafast laser ablation simulator can be realized using deep neural networks. The simulator can calculate the three-dimensional structure after irradiation by multiple laser pulses at arbitrary positions and with arbitrary pulse energies, and we applied the simulator to a variety of materials, including dielectrics, semiconductors, and an organic polymer. The simulator successfully predicted their depth profiles after irradiation by a number of pulses, even though the neural networks were trained using single-shot datasets. Our results indicate that deep neural networks trained with single-shot experiments are able to address physics with irreversibility and chaoticity that cannot be accessed using conventional repetitive experiments.

## Introduction

When an intense ultrafast laser pulse (typically lasting less than a picosecond = 10^−12^ s) is focused on a material, the rapid energy deposition from that pulse leads to material removal with little or no thermal degradation^1,2^. Irradiation using multiple laser pulses allows high-quality drilling, cutting, or microstructuring to be performed using optimized processing parameters. Currently, the planning and optimization of these processing parameters must be determined on a trial-and-error basis when using a real laser processing machine. The parameter space, which includes the beam trajectory, the pulse energy, the pulse duration, and the wavelength, is so vast that the optimization process takes considerable time and energy for each material and each application.

One of the significant challenges in this field is the construction of an effective model that can simulate spatial profiles after multiple-pulse irradiation to enable planning and optimization of the process parameters. There are three major difficulties in the model development. First, the physics that governs ultrafast laser ablation is still unclear and is a subject of intense debate^3–19^. The strong electric fields of laser pulses, which are comparable to the electric field inside an atom, drive target materials into states that are strongly out of equilibrium^3,11,15,19^. This situation contrasts with the case of material removal using much longer pulses, in which thermal effects, e.g., melting or vaporization, dominate the process^20^. Second, the relevant phenomena range in multi-spatiotemporal scales from femtosecond and angstrom to microsecond and micrometre. For each scale, computationally demanding calculations including first-principles calculations and molecular dynamics simulations are required^9,12,13,17,18,21^, and unification of these calculations is challenging.

Finally, pulse irradiation induces changes in the material properties including amorphization, defect formation and surface morphology, which could substantially alter the ablation process for subsequent pulses^10,14,16,22–25^.

Recent advances in machine learning technology have provided another approach: a way to build models from large datasets. In previous studies, the applications of machine learning methods such as neural networks to laser processing were reported^26–28^. Specifically, deep neural networks produce approximate functions from various types of inputs and outputs^29,30^ and can be used to extract hidden features from massive datasets^31,32^. B. Mills et al. used a generative adversarial network to predict laser-ablated surface profiles after three sequential pulse irradiation on an electroless nickel mirror^33–35^. In their studies, the neural network takes as input three beam profiles spatially modulated by a digital mirror device and outputs an image of the laser-ablated surface. While this method successfully reproduced the results of laser processing on a well-polished metal surface, it is not applicable for multiple-location irradiation and cannot be used for predicting micromachining with beam scanning.

Here, we developed a deep-neural-network-based laser-ablation simulator that can predict a three-dimensional depth profile after multiple-pulse irradiation on arbitrary locations for arbitrary pulse energies.

## Simulation architecture

**Figure 1:**
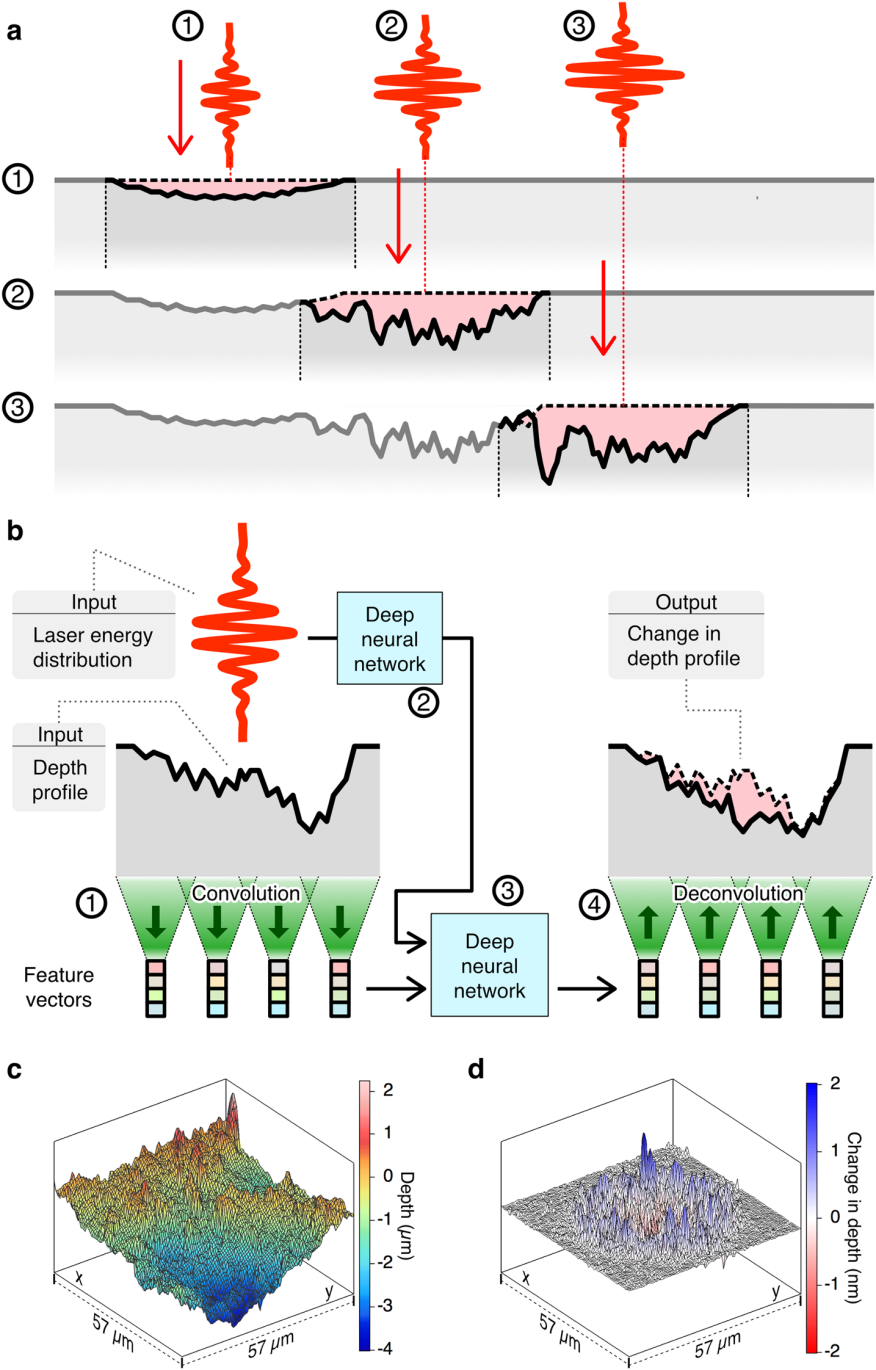
Schematic of laser ablation simulator using deep neural networks. (**a**) Simulation procedure. The simulator extracts a depth profile around an irradiation position as indicated by the vertical dotted lines and performs the calculation. The simulator calculates changes in the depth profiles caused by single-shot irradiation, represented by the pink shaded areas. The dashed lines and solid lines represent the depth profiles before and after irradiation, respectively. These calculations can be repeated at arbitrary irradiation positions using arbitrary pulse energies. (**b**) Configuration of the four deep neural networks. The first neural network performs a convolution to extract the feature vectors from the input depth profile. The second neural network calculates the responses to the incident laser pulse at each point. The third neural network combines the outputs from these two neural networks and calculates the nonlocal nonlinear interactions. The final neural network then performs a deconvolution to produce the output. (**c**,**d**) Three-dimensional representation of a typical training dataset. (**c**) Depth profile (96 × 96 pixels). (**d**) Changes in the depth profile (96 × 96 pixels).

Consider a situation where a series of ultrashort laser pulses are irradiated on to a target as shown in Fig. 1a. If we can calculate the changes in depth profiles that are induced by a laser pulse for arbitrary target depth profiles, we could also simulate the corresponding depth profiles after multiple-pulse irradiation. One of our major findings is that the depth profile that is formed by laser pulses has a characteristic morphology and this surface morphology provides sufficient information to predict changes in the depth profile caused by the next pulse. This means that we can extract the relevant material properties for the target from its local surface morphology using a deep neural network. Another finding is that highly nonlinear and nonlocal processes, including light-matter interactions and ablation, can also be expressed using deep neural networks. We formed the simulator by combining these neural networks with a final deep neural network that generated changes in the two-dimensional depth profiles that were induced by laser irradiation, as shown in Fig. 1b.

The simulator uses the following quantities as inputs to the deep neural networks: the depth profile before irradiation and the spatial energy distribution of the laser pulse. The simulator applies a set of convolutional neural networks to the input depth profile to produce a 128-dimensional feature vector at each point on a coarse-grained grid. The vectors that were deduced from the local surface morphology represent the relevant material properties, including the accumulated changes that were induced by preceding pulses. The subsequent neural networks calculate the interactions of these vectors with the input laser parameters through application of nonlinear transformations and the results are used to approximate the single-shot laser ablation process and produce the output (see “Methods” Section for full details).

We trained our deep neural networks via supervised learning using a set composed of a two-dimensional depth profile and its changes, as shown in Fig. 1c,d, respectively, along with the irradiating laser parameters. Depth profiles were acquired using our in-situ depth profile measurement system^25^ for target materials including silicon, sapphire, chemically strengthened glass and polyimide. We prepared thousands of datasets composed of two-dimensional depth profiles with nanometre-scale precision by irradiating each material with laser pulses at random positions and at random pulse energies (see “Methods” Section).

## Results for single-pulse irradiation

**Figure 2:**
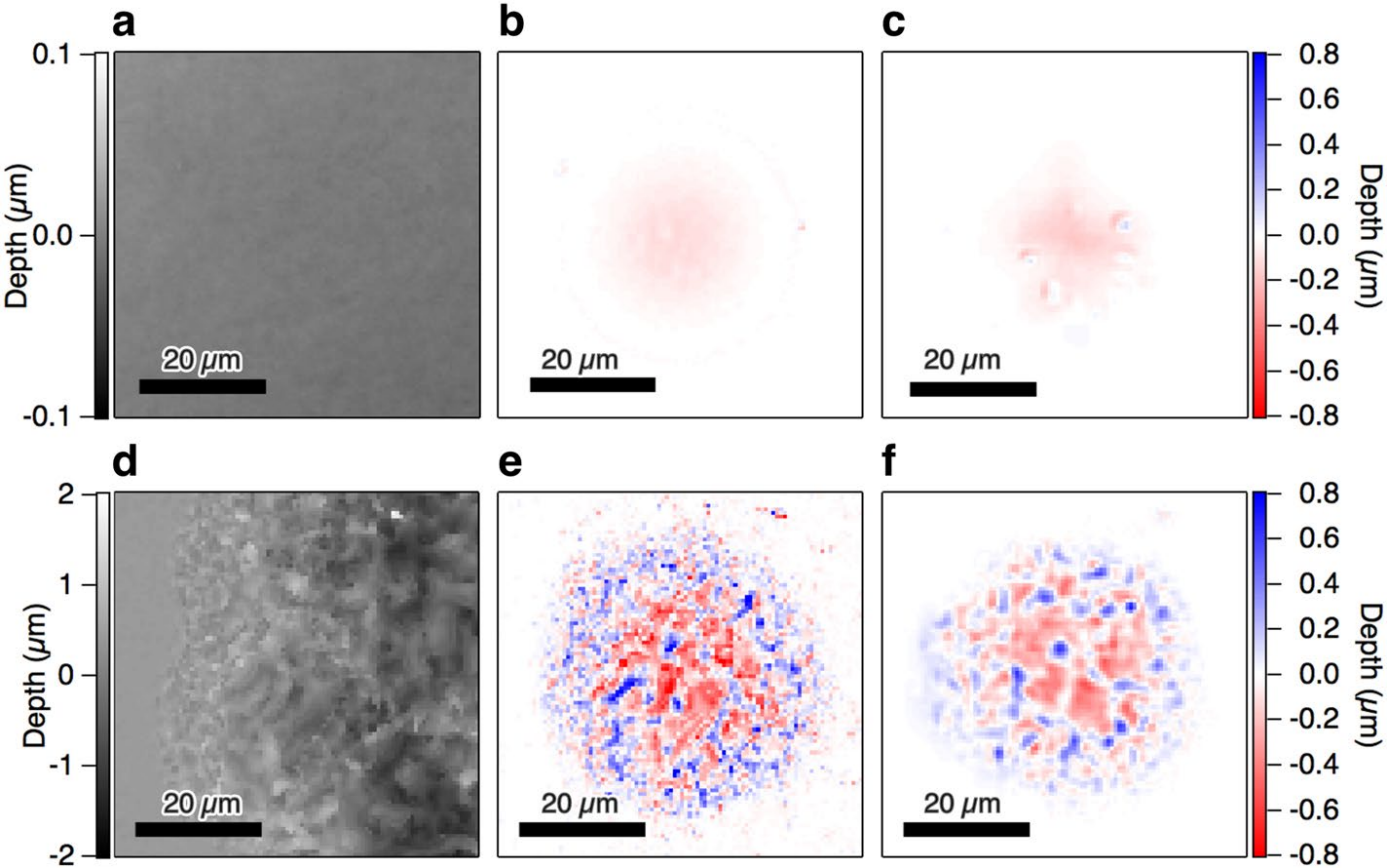
Comparison of experiments with simulations for single-shot irradiation. (**a**–**c**) Evaluation of an intact surface, (**d**–**f**) Evaluation of a laser-irradiated surface. (**a**,**d**) Depth profiles before irradiation. (**b**,**e**) Changes in depth profiles obtained experimentally. (**c**,**f**) Changes in depth profiles calculated using the simulator. A silicon substrate was used as the test target. The pulse energy for each evaluation was 40 mJ.

To evaluate the simulator, we compared depth profiles that were obtained experimentally with those from the simulations after single-pulse irradiation on intact and laser-irradiated surfaces, as shown in Fig. 2. We used a silicon substrate as the test target for this evaluation. Despite its industrial importance, there is no simulator for silicon that can predict the three-dimensional shape after multiple femtosecond pulse irradiation at present. This is due to several practical difficulties, including competing nonlinear optical absorption processes^36^ and the accumulated changes in the material properties during multiple-pulse irradiation^37^. The irradiating pulse energy was set at 40 mJ for each experiment and simulation. When the target surface is well-polished and intact, as shown in Fig. 2a, the changes in the depth profile induced by laser ablation are smooth, as shown in Fig. 2b, while those obtained for the surface after multiple-pulse irradiation are enhanced and bumpy, as shown in Fig. 2d,e. The differences should be the laser-induced changes in the material properties and the changes in the light-matter interactions. Figure 2c,f show the corresponding changes in the depth profiles that were calculated using the simulator for the depth profiles shown in Fig. 2a,d, respectively. The characteristics described above are reproduced well. The datasets used for the evaluation are not included among the datasets used for training throughout the paper (see “Methods” Section for the details). Note that our approach is not to aim for an exact match of the surface morphology, but to generate a surface that reproduces the features of the surface morphology. With this approach, we can use a deterministic difference equation based on the neural network to handle data containing chaotic processes in laser processing, such as resolidification and amorphization. The good agreement demonstrates that the feature vectors that were deduced from the local surface morphology play essential roles and that the deep neural networks can capture them. In other words, the surface morphology produced by the neural network contains enough information to predict the subsequent-pulse ablation. To further verify the predictability of the developed neural network, we perform quantitative evaluations of the simulator with respect to the primary objective, i.e., the depth profiles after multiple-pulse irradiation.

## Results for multiple-pulse irradiation

**Figure 3:**
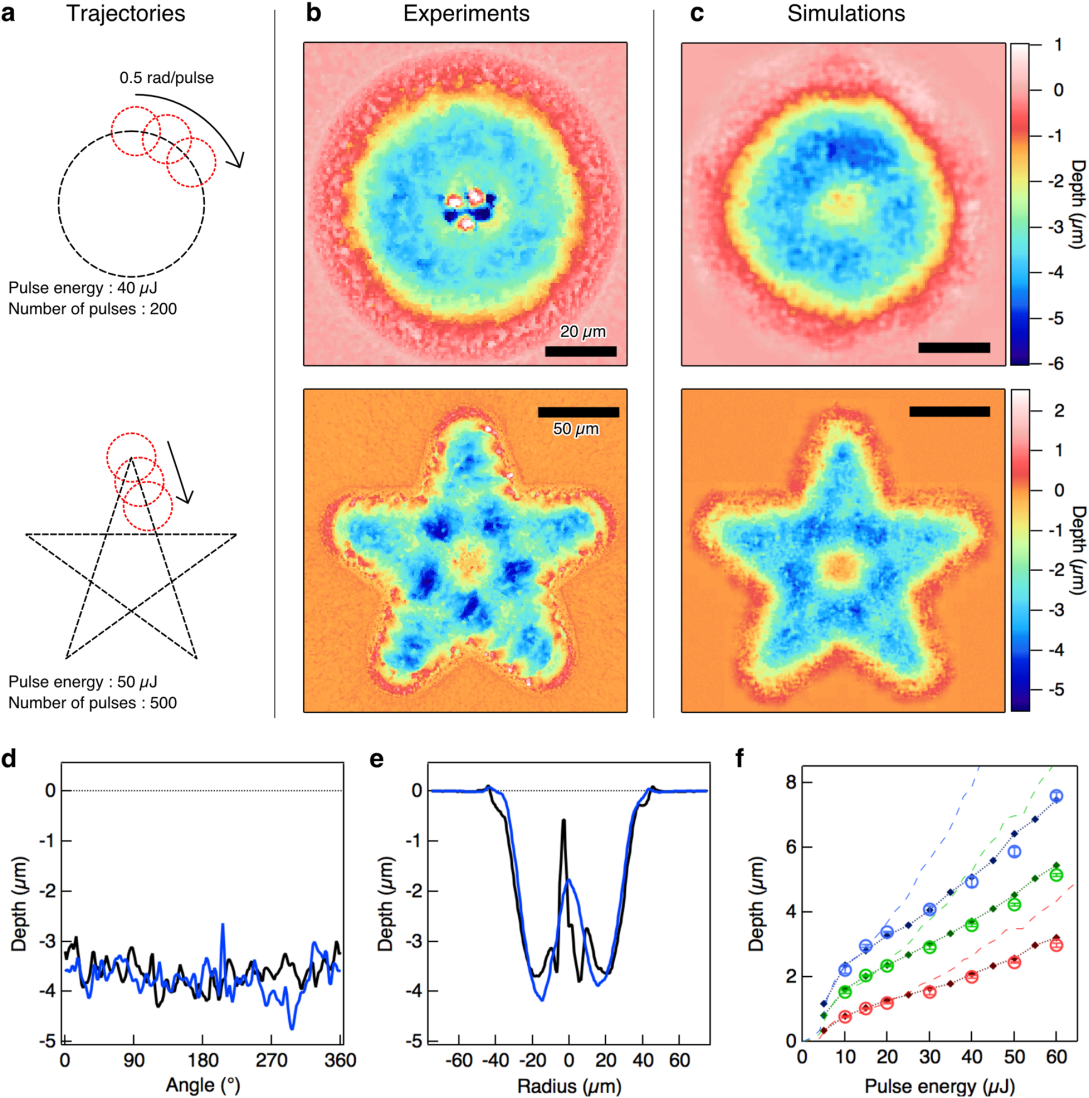
Comparison of experiments with simulations for multi-shot irradiation. (**a**) Trajectories for multi-shot irradiation. (**b**,**c**) Final depth profiles after irradiation obtained by experiments and simulations, respectively. (**d**,**e**) One-dimensional depth profiles on circular irradiation from the simulation (blue lines) and the experiment (black lines). (**d**) Depth profiles along the circular trajectory. (**e**) Depth profiles along the radial cross-section (see “Methods” Section). (**f**) Averaged ablated depth along the circular trajectory as a function of the pulse energy from the simulation (open circles) and the experiment (filled diamonds). Intact initial surfaces were irradiated using 100 (red), 200 (green), or 300 (blue) pulses. Error bars for the experimental results were calculated using the standard deviation from three independent measurements. Dashed lines represent the calculation reproduced from the arithmetic mean of the ablated depth over the training datasets for each of the pulse energies (see “Methods” Section). A silicon substrate was used as the test target.

We compared the depth profiles that were obtained from the simulations with those from the experiments after multiple-pulse irradiation, as shown in Fig. 3. Figure 3a shows the laser irradiation trajectory. The simulator calculated the two-dimensional depth profiles after laser irradiation along the circular or star-shaped trajectories using hundreds of pulses. The experimental verification was performed under the same irradiation conditions. Figure 3b,c show the resulting depth profiles that were obtained from the experiment and the simulation, respectively. The simulated results agree well with the experimental data without use of any adjustable parameters. For quantitative comparison, the depth profiles measured along the circular trajectory and those measured along the radial cross-section are plotted in Fig. 3d,e, respectively. The simulator reproduced both the average depth and the roughness of the depth profile well. This good agreement holds for a wide range of incident pulse energies, as illustrated in Fig. 3f, which shows the averaged ablated depth as a function of the pulse energy. The deep neural networks thus accurately reproduced these multi-shot ablation processes. One concern is that the neural network may just be outputting the fluence-dependent average ablation depth, in addition to random bumps proportional to the surface roughness before irradiation. To eliminate this possibility, the results of predicting the depth after multi-pulse irradiation based on the fluence-dependent average ablation depth in the training data are shown by dashed lines (see “Methods” Section). If the neural network were simply outputting an average value, the predictions should be on these dashed lines, but the neural network shows much better predictive performance. This demonstrates that the neural network has succeeded in extracting enough surface features to predict the outcome of the process, and in generating surface features with sufficient characteristics to estimate the changes caused by subsequent pulses. An example showing pulse-by-pulse comparison of the simulation results with the experimental measurements is provided in the Supplementary Information.

**Figure 4:**
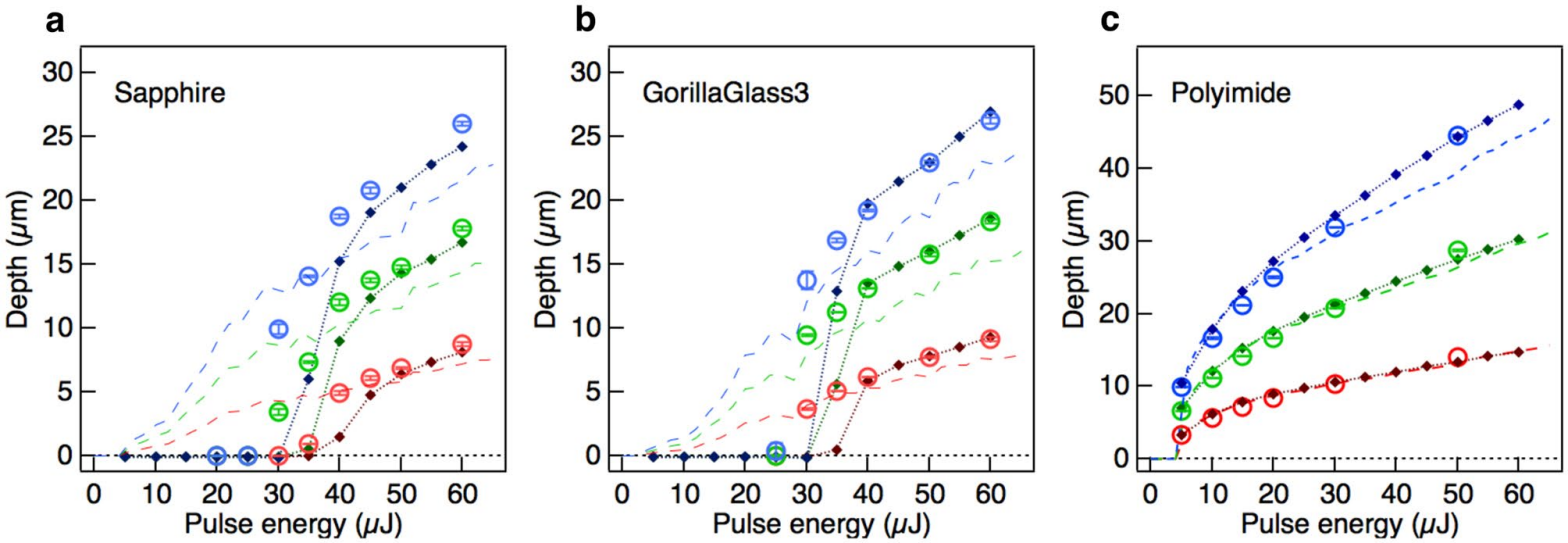
Comparison between experiments and simulations for various materials. Averaged ablated depths along the circular trajectory from the simulation (open circles) and the experiment (filled diamonds) are plotted as a function of the pulse energy. The radius of the circular irradiation is 20 mm. Intact initial surfaces were irradiated using 100 (red), 200 (green), or 300 (blue) pulses. The error bars for the experimental results were calculated using the standard deviation from three independent measurements. Dashed lines represent the calculation reproduced from the arithmetic mean of the ablated depth over the training datasets for each of the pulse energies (see “Methods” Section). (**a**), Sapphire. (**b**) Chemically strengthened glass (Corning Gorilla Glass 3). (**c**) Polyimide.

The simulator can be applied to a variety of materials that would otherwise require completely separate models. We trained the neural networks for each of the required materials, including sapphire, chemically strengthened glass, and an organic polymer film. Each material has a different band-gap, nonlinear optical coefficient, melting temperature, and other optical, thermal and mechanical properties. Each material is trained separately, but the structure of the network and the hyper parameters of the training are identical. Figure 4 shows the average depths along the circular trajectory as a function of the pulse energy, as per Fig. 3. The neural networks capture both the material-dependent nonlinearities and their cumulative behaviours. The deviation from the experimental results, particularly around the ablation thresholds, would be due to the limitations of the optical system in terms of its ability to capture the changes in the material properties.

## Conclusion

Our approach provides a quantitative method to treat phenomena with irreversibility and chaoticity. Our datasets consisted of vast numbers of pairs of an initial state and changes in that state, and almost none of the initial states are identical. This situation is in contrast to the conventional methodology in physics, which assumes an identical initial state before the response measurements when performing repetitive experiments.

## Methods

### Samples

We used (100)-silicon substrates, c-face sapphire substrates, chemically strengthened glass plates (Gorilla Glass 3, Corning), and polyimide films with surface roughnesses (*S*_*a*_) of 2 nm, 3 nm, 2 nm, and 70 nm, respectively. These surface roughnesses were measured using a white-light interferometric microscope (Contour GT-K, Bruker).

### Laser irradiation

We used a 1 kHz Ti:sapphire regenerative amplifier (Astrella, Coherent Inc.) as the light source. The centre wavelength of this laser was 800 nm, and the pulse duration was 50 fs. The focal length of the focusing lens was 150 mm, and the laser spot size at the focus position was 26 mm at the full width at half maximum, which was measured using the diameter regression technique.

### Training datasets

The training datasets were prepared using a pulse-by-pulse depth profile measurement system^25^. The system consists of a precision motorized stage, a white-light-interference microscope, and the optical system described above. The system compares the depth profiles before and after the laser pulse irradiation and can measure the change in depth profile by a single laser pulse irradiation with a precision of 3 nm. The acquired two-dimensional depth profiles consisted of 96 × 96 pixels, corresponding to dimensions of 57 × 57 mm (one pixel corresponds to 0.6 mm). For a series of data acquisition measurements, the system irradiated the target with 200–2000 pulses at random positions over a ring with an inner radius of 35 mm and an outer radius of 65 mm. The pulse energy was randomly varied over the range from 5 to 75 mJ for each pulse. The number of pulses is chosen to avoid situations where the total ablated depth exceeds 10 µm and the measurement time with the white-light-interference microscope becomes too long. Each single-shot acquisition took 20–40 s, including the data saving time. After the series of data acquisition measurements, the sample position was moved by 500 mm and another series of measurements was carried out using the same procedure. In this way, datasets on the changes in the depth profile caused by a laser pulse on a fresh surface and on a surface under the processing could be obtained. Datasets that included unmeasurable regions due to insufficient numbers of interferometric fringes were omitted. We prepared 39,000, 11,800, 8600, and 3700 training datasets for the silicon, sapphire, chemically strengthened glass, and polyimide materials, respectively.

### Testing datasets for single-pulse irradiation

The acquisition of the testing datasets for the single pulse irradiation, Fig. 2 and the supplementary data, was carried out in the same procedure as for the training data acquisition. The acquisition of testing datasets was done in a series completely independent of the acquisition of the dataset for training. They were carried out on different days and with different samples. The input of the neural network is 96 × 96 pixel, so even if the height profile has only two levels, high and low, there are 2^96 × 96^ > 10^2774^ patterns, which cannot be included in tens of thousands of training data almost certainly. The protrusions in Fig. 2c may appear to be a computational artefact, but this often happens in actual experiments as shown in Supplementary Fig. S3a. Such a structure is caused by the presence of dust on the surface before laser irradiation and is particularly noticeable on clean surfaces. These dusts were generated by laser irradiation during the previous data acquisition. They are visible under a brightfield microscope but is too small to be seen in a white-light-inteference 3D microscope. For a simulator looking only at a 3D surface, it is rather natural for characteristic protrusions to appear on a flat surface.

### Evaluation datasets for multiple-pulse irradiation

In the circular trajectory shown in Fig. 3, the radius of each of the circles was 20 mm and the angular frequency of irradiation was 0.5 rad/pulse. The pulse energy was set at 40 mJ and the surface was irradiated using 200 pulses. In the star-shaped trajectory, the length of each of the edges was 125 mm and the irradiation pitch was 12.5 mm. The pulse energy was set at 50 mJ and the surface was irradiated using 500 pulses. We stitched 3 × 3 two-dimensional depth profiles together to obtain an entire depth profile for the star-shaped depth profile. The pulse irradiation rate was 1 Hz for all datasets.

### Data processing of the evaluation datasets

We calculated the one-dimensional depth profile along the radial cross-section in Fig. 3e by averaging the radial cross-section from 0 to 180°. We then calculated the average of the ablated depth over the training datasets for each pulse energy, as represented by the dashed lines shown in Figs. 3f and 4, based on the arithmetic mean of the ablated depth over these training datasets and using the following formula:

$$\left\langle d \right\rangle = \frac{1}{N}\mathop \sum \limits_{i} \frac{1}{2\pi }\smallint d\theta \smallint dr d_{i} \left( {r,\theta } \right)/2\pi R,$$

where *N* represents the number of training datasets with a specific irradiation pulse energy, $d_{i} \left( {r,\theta } \right)$ represents the depth in polar coordinates (where the origin is at the irradiation position in the *i*th dataset), and *R* represents the radius of the circular irradiation used in the evaluation. Averaging is performed over every 2 mJ of the pulse energy. We multiplied $\left\langle d \right\rangle$ by the number of pulses with which the surface was irradiated to calculate the average ablated depth value over the training datasets.

### Deep neural networks and their training

The first neural networks (NNs) consisted of one normalization layer and three subsequent layers of convolutional NNs with a batch renormalization process and these NNs converted the input depth profile into feature vectors. The second NNs consisted of two average pooling layers and two fully-connected layers and these NNs calculated the local nonlinearities of the optical responses at each point on the grid. The third NNs consisted of a merging layer for the outputs from the first and second NNs and a U-net^38^ and these NNs calculated the nonlocal light-matter interactions. The final NNs consisted of deconvolutional NNs that were used to generate changes in the fine and coarse depth profiles. The sum of two changes in the depth profiles resulted in the output, i.e., the changes in the depth profile. The NN structures are described in detail in the Supplementary Information.

These NNs were constructed, trained and executed in Keras using Tensorflow as a backend.

For the training datasets, the input depth profiles and the laser energy distributions were passed directly to the deep NN, while the changes in the depth profiles were separated into two components; one contained the high spatial frequency components and the other contained the remaining coarse components. The high-frequency spatial filter applied used a box average over 8 × 8 pixels.

### Data augmentation for training

We performed data augmentation to allow the neural networks to acquire the following traits: the output, i.e., the changes in the depth profile, should be insensitive to the absolute heights of the input depth profile and should be zero when the incident pulse energy is zero. The following two types of datasets were added: datasets with homogeneous offsets in their initial depth profiles and datasets with zero pulse energy and no changes in their depth profiles. In the former augmentation, a uniform random number in the range of − 1 to + 1 mm was added as an offset to the input depth profile, while the pulse energy and output were the same. This data augmentation increased the number of datasets by a factor of four. The latter augmentation set the input pulse energy to zero and the output differential depth profile to zero, while the input depth profile was the same. This data augmentation increased the number of datasets by a factor of two.

## Supplementary Information

Supplementary Information 1.

Supplementary Video 1.

## Data Availability

The datasets generated and/or analysed during the current study are not publicly available due to their huge data size (more than 1 TB) but are available from the corresponding author on reasonable request.

## Code availability

The neural network models described above were written in Python and are available from the corresponding author upon reasonable request.
