# Supplementary material for: Ultrafast laser ablation simulator using deep neural networks
Source: Sci Rep. 2022 Apr 7;12:5837. doi: 10.1038/s41598-022-09870-x (PMC8990072; doi:10.1038/s41598-022-09870-x)
Supplement: Supplementary file 1 — Supplementary Information 1. [file 41598_2022_9870_MOESM1_ESM.pdf]

Supplementary Materials

Ultrafast laser ablation simulator using deep neural networks

Shuntaro Tani[1,*] , Yohei Kobayashi[1]

[1]The Institute for Solid State Physics, The University of Tokyo, Kashiwa, Chiba 277-8581, Japan.

Structures and parameters of neural networks used in our simulator

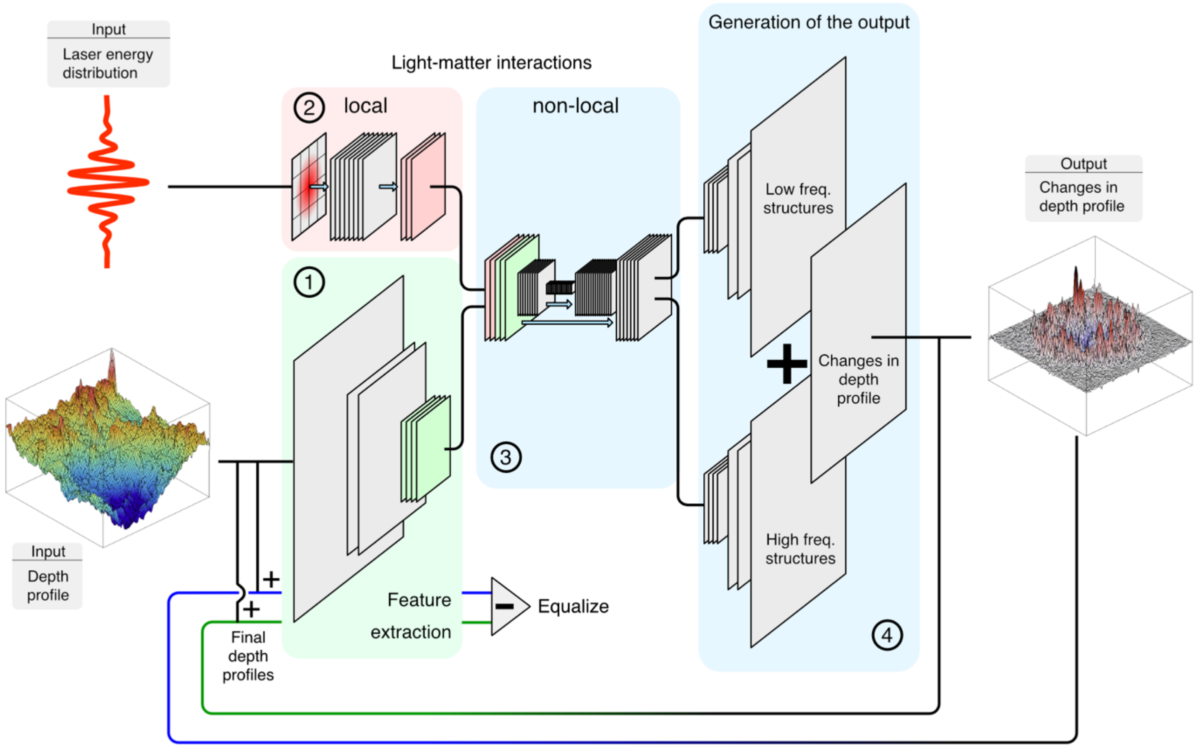

Fig. S1. Schematic structures of the neural networks used in this study. The ordering of the neural networks correspond to those in Fig. 1 and Methods. The first neural network performs a convolution to extract the feature vectors from the input depth profile. The second neural network calculates the response to the incident laser pulse at each point. The third neural network combines the outputs from these two neural networks and calculates the nonlocal nonlinear interactions. The final neural network then performs a deconvolution to produce the output.

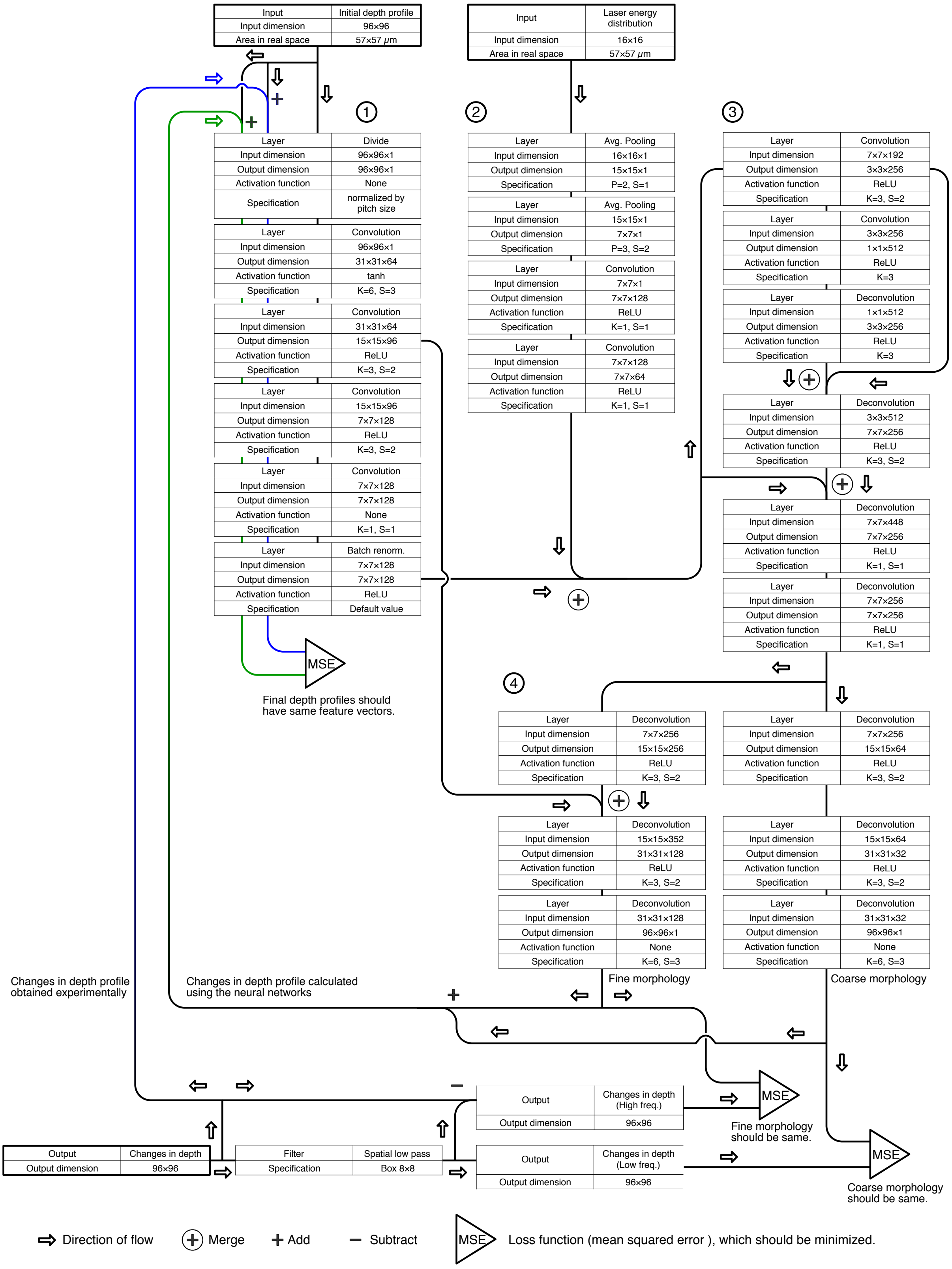

Fig. S2. Structures and parameters of the neural networks in detail. The numbers of neural networks correspond to those in Fig. S1. K represents kernel size, or the height and width of a 2D convolutional window. S represents a step size of a convolution along the height and width. Supervised learning simultaneously minimizes the loss functions, which are represented by triangles.

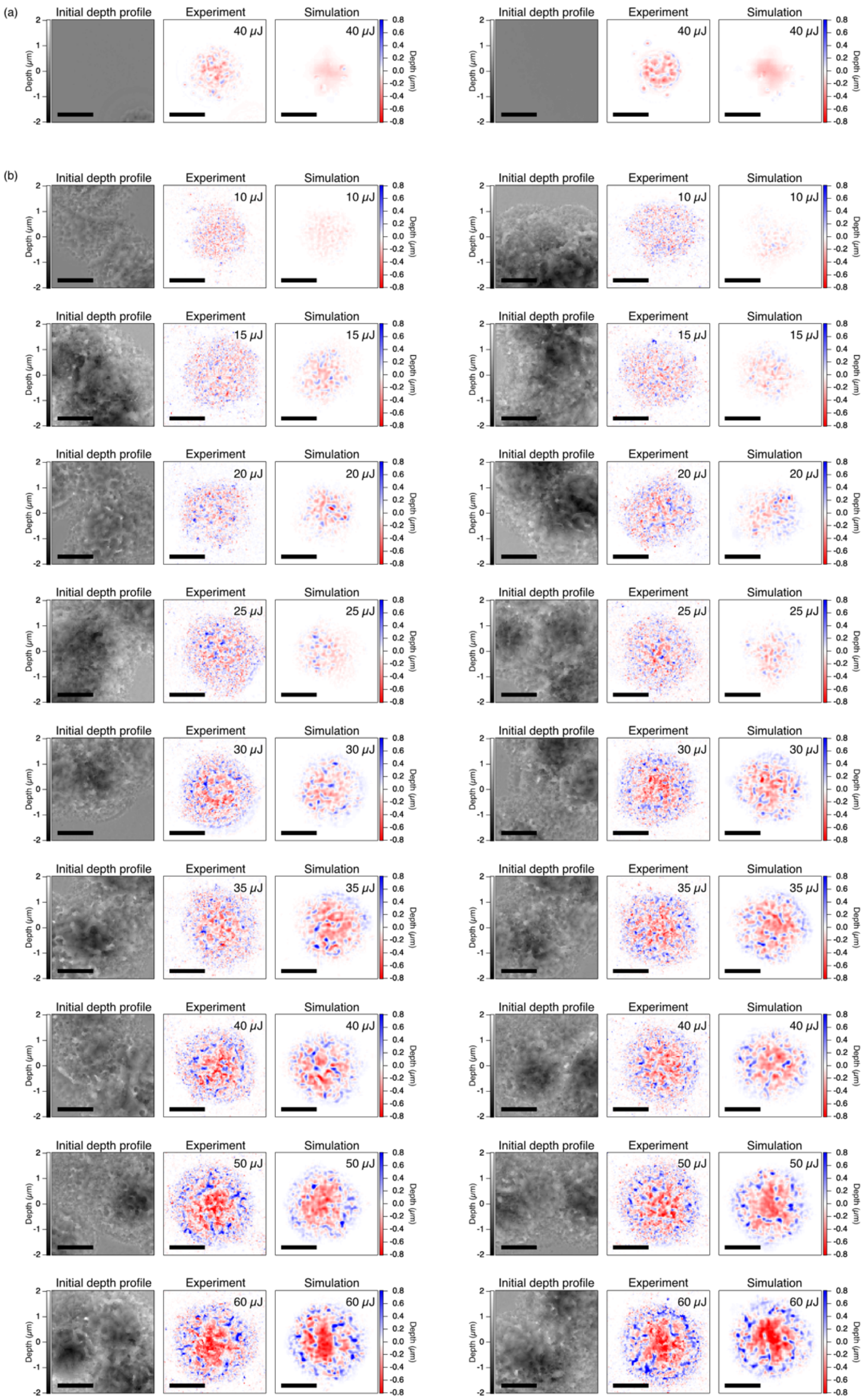

Fig. S3. Single-shot prediction examples for silicon. Differences in depth profiles obtained experimentally for various initial morphologies with a single shot, and simulated results for the same initial morphologies with the same pulse energy. All validation data are obtained in separate experimental processes from the training data and are completely independent of the training data. (a) Protrusions and pits induced by dust when a single laser shot is applied to a clean surface. The dusts are so small that they are not visible in the initial morphologies and therefore hardly appear in the simulation results. (b) Predictions for uneven surface features created by multiple laser pulses.
